# Assessment of Hypercoagulability After Orthopedic Trauma Surgery Using Coagulation Biomarkers and TEG/ROTEM

**DOI:** 10.3390/ijms27156939

**Published:** 2026-08-02

**Authors:** Bogdan Huzum, Dan Viorel Cionca, Daniel Ciupilan, Dragos-Florin Tesoi, Cornelia Mircea, Oana-Viola Badulescu

**Affiliations:** 1 Grigore T. Popa University of Medicine and Pharmacy, 700115 Iasi, Romania; bogdan.huzum@umfiasi.ro (B.H.); cornelia.mircea@umfiasi.ro (C.M.); oana.badulescu@umfiasi.ro (O.-V.B.); 2 Orthopedic Clinic, “St. Spiridon” County Emergency Clinical Hospital, 700111 Iasi, Romania; dancionca@yahoo.com (D.V.C.); dciupilan@gmail.com (D.C.); 3 Emergency Clinical Hospital for Children “Sfanta Maria”, 700309 Iasi, Romania; 4 Hematology Clinic, “St. Spiridon” County Emergency Clinical Hospital, 700111 Iasi, Romania

**Keywords:** thromboelastography, rotational thromboelastometry, thrombin–antithrombin complexes, D-dimer, fibrinogen, total hip arthroplasty, total knee arthroplasty

## Abstract

Orthopedic trauma surgery is associated with an increased risk of postoperative hypercoagulability, predisposing patients to potentially life-threatening thromboembolic complications, including deep vein thrombosis (DVT) and pulmonary embolism (PE). Conventional coagulation tests, including prothrombin time (PT), activated partial thromboplastin time (aPTT), and international normalized ratio (INR), have limited sensitivity in detecting the complex hemostatic alterations that occur following orthopedic trauma procedures. In recent years, viscoelastic hemostatic assays—thromboelastography (TEG) and rotational thromboelastometry (ROTEM)—alongside specific coagulation biomarkers, such as D-dimer, fibrinogen, thrombin–antithrombin (TAT) complexes, and prothrombin fragments F1+2, have emerged as promising tools for a more comprehensive assessment of coagulation status in the perioperative setting. This narrative review, conducted using a structured literature search strategy with systematic elements, aims to synthesize the current evidence regarding the use of TEG/ROTEM and coagulation biomarkers in evaluating hypercoagulability following orthopedic trauma surgery, identify patterns of hemostatic dysregulation across different surgical procedures and patient populations, and highlight existing gaps in the literature. A structured search was conducted across major electronic databases, including PubMed, Scopus, Web of Science, Embase, and the Cochrane Library. The findings of this review provide clinicians with a clearer framework for perioperative coagulation monitoring and inform future research aimed at optimizing individualized thromboprophylaxis strategies in orthopedic trauma patients.

## 1. Introduction

Venous thromboembolism (VTE), encompassing deep vein thrombosis (DVT) and pulmonary embolism (PE), remains one of the most serious and potentially life-threatening complications following orthopedic trauma surgery [1]. Patients undergoing major lower-extremity orthopedic procedures—including total hip arthroplasty (THA), total knee arthroplasty (TKA), and hip fracture surgery—are considered among the highest-risk surgical populations for VTE development [2]. In the absence of pharmacological thromboprophylaxis, rates of venographically detected DVT reach 54% after THA and up to 64% after TKA, while fatal PE has been reported in up to 2.0% of patients undergoing total hip arthroplasty and up to 7.5% of those undergoing hip fracture surgery [3,4]. Even with contemporary anticoagulant prophylaxis, VTE still occurs in an estimated 4.4% of patients undergoing major arthroplasty procedures, with the greatest risk concentrated within the first 7 to 14 days postoperatively [5,6].

The pathophysiology of postoperative hypercoagulability in orthopedic trauma patients is multifactorial, encompassing all three components of Virchow’s triad: endothelial injury caused by the surgical procedure itself, venous stasis secondary to immobilization and tourniquet use, and a systemic prothrombotic state driven by the activation of the coagulation cascade [7]. This hypercoagulable state begins immediately after tissue injury. Tissue factor release, platelet activation, thrombin generation, and fibrin deposition occur early and persist throughout the perioperative period [4,8].

Despite decades of research, the accurate and timely identification of patients at highest thrombotic risk remains a clinical challenge. Conventional coagulation tests—including prothrombin time (PT), activated partial thromboplastin time (aPTT), and international normalized ratio (INR)—reflect only isolated components of the coagulation cascade under static, plasma-based conditions and have demonstrated limited sensitivity in detecting the complex hemostatic alterations that characterize postoperative hypercoagulability [9]. These assays fail to account for the contributions of platelets, factor XIII, and the fibrinolytic system, thereby providing an incomplete picture of the patient’s true coagulation status [9].

In response to these limitations, viscoelastic hemostatic assays (VHAs)—namely thromboelastography (TEG) and rotational thromboelastometry (ROTEM)—have emerged as valuable tools for the global, dynamic evaluation of coagulation in whole blood [10]. Unlike conventional tests, TEG and ROTEM capture the full hemostatic process, from initial clot formation through clot strength and eventual fibrinolysis, providing real-time, clinically actionable data [10,11]. A systematic review and meta-analysis by Brown et al. demonstrated that TEG—particularly the maximum amplitude (MA) parameter—consistently detected hypercoagulability starting from postoperative day 1 in orthopedic surgical patients, although significant variability in the definition and cut-off values for hypercoagulability was noted across studies [12]. Furthermore, Tsantes et al. demonstrated that several abnormal ROTEM parameters were significantly associated with the presence and development of symptomatic VTE after hip fracture surgery, highlighting the potential clinical value of these assays in perioperative risk stratification [13]. Together, these studies suggest that VHAs may identify postoperative hypercoagulability earlier than conventional coagulation assays while providing clinically relevant information regarding thrombotic risk.

Alongside viscoelastic testing, specific coagulation biomarkers—including D-dimer, fibrinogen, thrombin–antithrombin (TAT) complexes, prothrombin fragments F1+2, and plasminogen activator inhibitor-1 (PAI-1)—have been investigated as markers of coagulation activation and fibrinolytic impairment in the orthopedic perioperative setting [14,15,16]. However, the optimal combination of biomarkers and the most informative temporal windows for their measurement remain poorly defined, and no validated algorithm currently integrates VHA findings with biochemical markers to guide individualized thromboprophylaxis decisions [12].

Given the growing body of evidence supporting the utility of TEG/ROTEM and coagulation biomarkers in this context, and considering the heterogeneity and fragmentation of the existing literature, a comprehensive synthesis of the available evidence is warranted. This narrative review aims to synthesize the available evidence regarding the use of TEG, ROTEM, and coagulation biomarkers in the assessment of hypercoagulability following orthopedic trauma surgery; to identify patterns of hemostatic dysregulation across different surgical procedures and patient populations; to characterize the methodological approaches and temporal profiles used in published studies; and to delineate existing knowledge gaps to inform future prospective research and clinical guidelines. From a clinical perspective, identifying patients who remain hypercoagulable despite standard thromboprophylaxis could enable individualized anticoagulant strategies and potentially reduce preventable postoperative VTE.

Although this review focuses primarily on orthopedic trauma surgery, much of the current evidence regarding perioperative hypercoagulability, coagulation biomarkers, and viscoelastic hemostatic assays has been generated in patients undergoing elective total hip and total knee arthroplasty. Because major orthopedic trauma surgery and elective arthroplasty share fundamental mechanisms of tissue injury, coagulation activation, endothelial dysfunction, and postoperative venous thromboembolism, evidence from both settings was incorporated where appropriate. Throughout the manuscript, findings derived from elective arthroplasty are interpreted within this context, while emphasizing the need for additional studies specifically conducted in orthopedic trauma populations.

## 2. Literature Search

A structured literature search was conducted across five major electronic databases: PubMed/MEDLINE, Scopus, Web of Science (Core Collection), Embase, and the Cochrane Library. The search covered publications from January 2000 to December 2024, with no language restrictions applied during the initial search phase. The following combination of Medical Subject Headings (MeSH) terms and free-text keywords was employed across all databases: orthopedic surgery OR orthopedic trauma OR hip fracture OR arthroplasty OR fracture fixation) AND (hypercoagulability OR coagulopathy OR venous thromboembolism OR deep vein thrombosis OR pulmonary embolism) AND (thromboelastography OR TEG OR rotational thromboelastometry OR ROTEM OR viscoelastic test OR D-dimer OR fibrinogen OR thrombin–antithrombin OR coagulation biomarker.

In addition to database searching, reference lists of all retrieved articles were manually screened to identify additional relevant studies not captured by the electronic search. Gray literature sources, including ClinicalTrials.gov, were also consulted.

All identified records were imported and managed using Rayyan (Qatar Computing Research Institute, Doha, Qatar), a validated web-based platform for literature screening [17]. After the removal of duplicates, titles and abstracts were screened independently against the following eligibility criteria. Studies were included if they: (1) involved adult patients (≥18 years) undergoing orthopedic trauma surgery; (2) reported perioperative or postoperative data on TEG, ROTEM, or at least one specific coagulation biomarker (D-dimer, fibrinogen, TAT complexes, F1+2, PAI-1, or von Willebrand factor); and (3) were published as full-text original articles, systematic reviews, or narrative reviews in peer-reviewed journals. Case reports, conference abstracts without full-text availability, and studies reporting exclusively standard coagulation parameters (PT, aPTT, INR) were excluded.

The selected literature was subsequently synthesized narratively and organized thematically into the following domains: pathophysiology of post-traumatic hypercoagulability, coagulation biomarkers, viscoelastic hemostatic assays (TEG and ROTEM), integration of biomarker and VHA findings, and clinical implications for thromboprophylaxis.

Because trauma-specific evidence remains limited, studies involving major elective orthopedic procedures (particularly THA and TKA) were also considered when they addressed shared mechanisms of perioperative hypercoagulability or the clinical performance of coagulation biomarkers and viscoelastic assays.

## 3. Pathophysiology of Post-Traumatic Hypercoagulability

### 3.1. From Virchow’s Triad to Immunothrombosis: An Updated Conceptual Framework

The pathophysiology of VTE in orthopedic trauma surgery is classically framed through Virchow’s triad (Table 1)—the tripartite model describing the interplay between venous stasis, endothelial injury, and hypercoagulability as the fundamental determinants of thrombus formation [7,18].

First conceptualized in the nineteenth century, this model has retained its clinical relevance and continues to serve as the theoretical backbone for understanding thrombogenesis across surgical and medical contexts. In the setting of orthopedic trauma, all three components of the triad are simultaneously activated, creating a uniquely hostile hemostatic environment that predisposes patients to both deep vein thrombosis (DVT) and pulmonary embolism (PE) [7,19].

Although Virchow’s triad remains the classical conceptual framework for understanding venous thrombosis, it no longer fully explains the molecular mechanisms responsible for trauma-induced hypercoagulability. While venous stasis, endothelial injury, and hypercoagulability continue to represent the major pathophysiological domains of thrombosis, contemporary evidence demonstrates that orthopedic trauma initiates a highly coordinated process of immunothrombosis, in which activation of the innate immune system is tightly integrated with coagulation. Rather than acting as independent processes, inflammation and coagulation form a bidirectional network that limits tissue damage but may also promote pathological thrombus formation when excessively activated [20,21].

Following orthopedic trauma, extensive disruption of bone, muscle, and vascular tissues results in the release of damage-associated molecular patterns (DAMPs), including extracellular DNA, histones, mitochondrial DNA, and high-mobility group box-1 (HMGB1). The magnitude of tissue injury influences the extent of DAMP release and the subsequent activation of the contact pathway, thereby modulating thrombin generation during the early post-traumatic response. These endogenous danger signals activate neutrophils, platelets, monocytes, and endothelial cells through pattern-recognition receptors, thereby initiating both inflammatory and procoagulant responses. Complement activation products, particularly C3a and C5a, further amplify this response by priming neutrophils for NETosis, thereby strengthening the mechanistic link between inflammation and immunothrombosis. Activated neutrophils subsequently undergo NETosis, releasing neutrophil extracellular traps (NETs), which consist of decondensed chromatin decorated with histones, neutrophil elastase, myeloperoxidase, and other antimicrobial proteins. Beyond their antimicrobial role, NETs provide a highly thrombogenic scaffold that promotes platelet adhesion, activation of factor XII, thrombin generation, fibrin deposition, and stabilization of the developing thrombus [20,22,23].

Platelets actively participate in this process beyond their traditional hemostatic function. Activated platelets express P-selectin and release inflammatory mediators that interact with PSGL-1 expressed on neutrophils, promoting reciprocal platelet–neutrophil activation and further NET formation. In parallel, activated platelets externalize phosphatidylserine, providing the catalytic membrane surface required for assembly of the factor Xa–factor Va (prothrombinase) complex, thereby markedly enhancing local thrombin generation. This positive feedback loop amplifies thrombin generation, reinforces fibrin accumulation, and contributes to the propagation of venous thrombosis. Simultaneously, activated endothelial cells lose their physiological anticoagulant phenotype while increasing the expression of adhesion molecules, tissue factor, and von Willebrand factor, thereby facilitating leukocyte recruitment and additional platelet activation. Consequently, hypercoagulability following orthopedic trauma should be viewed as the result of coordinated interactions between coagulation, innate immunity, circulating blood cells, and the vascular endothelium rather than activation of the coagulation cascade alone [20,24,25,26,27].

Therefore, the modern concept of immunothrombosis does not replace Virchow’s triad but substantially extends it by providing the molecular mechanisms linking tissue injury with systemic thrombin generation and venous thromboembolism. Within this updated framework, Virchow’s triad represents the clinical phenotype of thrombosis, whereas immunothrombosis explains the cellular and molecular events responsible for its initiation, amplification, and persistence after orthopedic trauma.

### 3.2. Damage-Associated Molecular Patterns and Contact Pathway Activation

Orthopedic trauma induces the extensive disruption of bone, muscle, and vascular tissues, leading to the rapid extracellular release of damage-associated molecular patterns (DAMPs), including mitochondrial DNA (mtDNA), high-mobility group box-1 (HMGB1), extracellular histones, ATP, and fragmented nuclear DNA. Unlike pathogen-associated molecular patterns (PAMPs), DAMPs originate from injured host cells and initiate sterile inflammation through the activation of pattern-recognition receptors, including Toll-like receptors (TLRs), the receptor for advanced glycation end products (RAGE), and intracellular DNA-sensing pathways. Increasing evidence indicates that these endogenous danger signals not only amplify inflammation but also directly promote coagulation, thereby providing a mechanistic link between tissue injury and trauma-induced hypercoagulability [28,29,30]. Beyond their immunomodulatory effects, several DAMPs activate the contact system, a pathway that remains underrepresented in many descriptions of trauma-associated coagulation. Extracellular DNA, mitochondrial DNA, RNA, polyphosphates, and histone–DNA complexes provide negatively charged surfaces that facilitate factor XII (FXII) autoactivation, initiating the intrinsic coagulation pathway. Activated FXII (FXIIa) subsequently converts prekallikrein into kallikrein and activates factor XI, creating a positive amplification loop that markedly enhances thrombin generation independently of the initial tissue factor stimulus. This contact pathway acts synergistically with the tissue factor pathway, sustaining coagulation even after the early burst of tissue factor exposure has diminished [30,31]. HMGB1 further amplifies this thromboinflammatory response through multiple complementary mechanisms. Binding of HMGB1 to TLR2, TLR4, and RAGE promotes endothelial activation, increases tissue factor expression on monocytes, enhances platelet activation, and facilitates neutrophil recruitment. Simultaneously, mitochondrial DNA released after orthopedic trauma activates innate immune signaling pathways because of its bacterial evolutionary origin, promoting neutrophil activation, platelet–leukocyte interactions, and NET formation, thereby reinforcing both contact pathway activation and thrombin generation. Rather than acting as isolated mediators, HMGB1 and mtDNA participate in a self-amplifying thromboinflammatory network that links sterile tissue injury with sustained coagulation activation [28,29]. Although tissue factor-mediated activation of the extrinsic pathway remains the dominant trigger immediately after orthopedic injury, accumulating evidence suggests that DAMP-mediated contact pathway activation is a major amplification mechanism responsible for maintaining thrombin generation during the subsequent inflammatory phase. This integrated model helps explain why hypercoagulability frequently persists beyond the initial surgical insult and highlights that trauma-induced coagulation results from coordinated activation of both the extrinsic and intrinsic pathways rather than tissue factor signaling alone [30,31].

### 3.3. Venous Stasis

Venous stasis—the slowing or interruption of physiological blood flow—represents one of the most prominent risk factors in the perioperative orthopedic setting. During surgical procedures such as total hip arthroplasty (THA) or total knee arthroplasty (TKA), patient positioning, intraoperative limb manipulation, and the use of pneumatic tourniquets directly impair venous return from the lower extremities [32]. Postoperatively, obligatory immobilization secondary to pain, wound care constraints, and protective weight-bearing restrictions further prolong stasis beyond the intraoperative window. In elderly patients with baseline venous insufficiency, this phenomenon is compounded by reduced muscular pump activity and impaired valvular competence of the deep venous system [7,19]. Reduced blood flow velocity disrupts the normal laminar flow pattern, promotes platelet–endothelium contact, and impairs the dilution and hepatic clearance of activated clotting factors—all conditions permissive for fibrin clot formation [18].

### 3.4. Endothelial Injury

Direct and indirect vascular endothelial injury constitutes the second pillar of thrombogenesis in orthopedic trauma. Surgical incision, retractor-induced compression, intramedullary reaming, and cement pressurization during arthroplasty all produce mechanical disruption of the vascular endothelium [33,34]. Upon injury, endothelial cells lose their constitutive antithrombotic properties—including the expression of thrombomodulin, prostacyclin, and tissue plasminogen activator (t-PA)—and shift toward a prothrombotic phenotype characterized by tissue factor (TF) expression, von Willebrand factor (vWF) release, and platelet adhesion molecule upregulation [35]. This endothelial phenotypic switch facilitates the propagation of coagulation beyond the site of injury and contributes to the transition from local hemostatic activation to systemic hypercoagulability. Of particular clinical relevance is the phenomenon of intramedullary pressurization during cemented arthroplasty: insertion of the femoral component forces bone marrow contents, including tissue thromboplastin, into the perifemoral venous plexus, directly activating the extrinsic coagulation cascade and generating thrombin [33,34]. In hip fracture surgery specifically, the combination of traumatic soft tissue disruption and subsequent surgical exposure generates a sustained endothelial activation state that persists well into the postoperative period [36].

### 3.5. Hypercoagulability and the Inflammatory–Coagulation Interface

The third component of Virchow’s triad—systemic hypercoagulability—arises from a complex interplay between coagulation cascade activation, inflammatory cytokine release, and fibrinolytic suppression in response to tissue trauma [37]. Tissue injury triggers the exposure of TF on activated monocytes, macrophages, and damaged endothelial cells, initiating the extrinsic pathway and culminating in a thrombin burst with subsequent fibrin deposition [37]. Simultaneously, bone marrow and soft tissue trauma releases pro-inflammatory cytokines—most notably interleukin-6 (IL-6) and tumor necrosis factor-alpha (TNF-α)—which amplify TF expression, induce acute-phase protein synthesis (including fibrinogen and CRP), and further suppress endogenous anticoagulant pathways [36,38]. In the orthopedic trauma context, this inflammatory–coagulation crosstalk manifests as a perioperative hypercoagulable state detectable from as early as the first postoperative day, and sustained for up to 14–21 days depending on the magnitude of the intervention [12]. The fibrinolytic system is simultaneously impaired: plasminogen activator inhibitor-1 (PAI-1)—the principal endogenous inhibitor of fibrinolysis—is upregulated in response to surgical stress and elevated IL-6 levels, further consolidating the net prothrombotic balance [14,39]. Concurrently, natural anticoagulant proteins such as antithrombin III and protein C are consumed or downregulated during the acute phase response, further shifting the hemostatic equilibrium toward thrombosis [37]. The net result is a perioperative coagulation phenotype characterized by increased thrombin generation (reflected by elevated thrombin–antithrombin [TAT] complexes and prothrombin fragments F1+2), elevated fibrinogen and D-dimer, and viscoelastic changes consistent with hypercoagulability on TEG and ROTEM testing [12,16]. Importantly, this state is not uniformly captured by standard coagulation tests such as PT, aPTT, and INR, which assess isolated pathway activity under non-physiological conditions and fail to reflect the global, whole-blood hemostatic picture—a fundamental limitation that underscores the growing interest in functional hemostatic assays and targeted biomarker monitoring in this population. The major VTE risk factors in orthopedic trauma, together with their underlying mechanisms, clinical consequences, and relevant biomarkers, are summarized in Table 2.

## 4. Coagulation Biomarkers—Evidence and Clinical Relevance

### 4.1. Overview: From Standard Tests to Targeted Biomarkers

The assessment of coagulation status in orthopedic trauma patients has historically relied on conventional parameters—prothrombin time (PT), activated partial thromboplastin time (aPTT), and international normalized ratio (INR). While these tests provide useful information about isolated pathway function, they assess coagulation under non-physiological, cell-free conditions and fail to capture the dynamic, whole-blood hemostatic processes that underlie perioperative hypercoagulability [37]. PT and aPTT were primarily designed to detect coagulation factor deficiencies by measuring the time to initial fibrin formation. Because clot formation occurs after only a small fraction of total thrombin has been generated, these assays do not capture the subsequent amplification phase of thrombin generation that characterizes many hypercoagulable states [40,41]. Importantly, these tests are systematically insensitive to the procoagulant changes most relevant in orthopedic surgery: activation of the extrinsic pathway, thrombin generation, fibrinolytic suppression, and platelet–fibrin interaction. This fundamental limitation has driven increasing research interest in a panel of targeted coagulation biomarkers capable of reflecting the full spectrum of hemostatic activation in the perioperative period [14,42].

### 4.2. D-Dimer: The Most Studied Biomarker

D-dimer—a fibrin degradation product generated by plasmin-mediated clot lysis—represents the most extensively investigated biomarker in the context of perioperative VTE risk in orthopedic surgery [43]. In the general clinical setting, D-dimer demonstrates high sensitivity (>95%) for VTE exclusion at the conventional cutoff of 0.5 mg/L, making it a valuable negative predictive tool. However, its specificity is substantially compromised in the postoperative orthopedic context, where surgical trauma, wound healing, and immobilization independently elevate D-dimer levels irrespective of thrombotic events [44,45]. Studies in patients undergoing THA and TKA have reported that 92–100% of patients remain above the conventional D-dimer threshold even at 6 weeks postoperatively, limiting the utility of the standard cutoff for VTE diagnosis [46]. To address this limitation, several groups have investigated procedure-specific and age-adjusted cutoff values. Lin et al. analyzed D-dimer in 10,775 fracture patients and demonstrated that the biomarker’s diagnostic yield improves substantially when combined with fibrinogen, particularly in patients over 60 years [43]. In hip fracture patients specifically, a proposed age-adjusted formula (age × 0.02 mg/L) has demonstrated improved specificity for preoperative DVT exclusion in elderly patients compared to the fixed standard cutoff [47]. Serial postoperative measurements of D-dimer have been investigated as a strategy to improve VTE detection following major orthopedic surgery, as marker levels evolve dynamically during the postoperative period [14,46]. Importantly, early postoperative D-dimer elevation primarily reflects physiological fibrin formation and degradation associated with surgical tissue repair rather than pathological thrombosis per se. Consequently, isolated D-dimer values obtained shortly after orthopedic trauma or surgery should be interpreted cautiously and always within the appropriate clinical context [45,46]. Despite these advances, D-dimer remains an indirect, non-specific marker whose perioperative utility is context-dependent and must be interpreted within a clinical framework that accounts for patient age, type of surgery, timing of measurement, and concurrent thromboprophylaxis [44,45]. Its greatest clinical value in orthopedic trauma likely lies not as a standalone diagnostic test, but as a component of multimarker prediction models.

### 4.3. Fibrinogen: Acute-Phase Reactant and Prothrombotic Substrate

Fibrinogen—the primary substrate for thrombin-mediated clot formation and a major determinant of clot mechanical strength—occupies a dual role in the perioperative hemostatic balance: it is simultaneously an acute-phase protein elevated in response to inflammation and a direct contributor to the thrombotic substrate [48]. In orthopedic trauma patients, fibrinogen levels increase progressively from the day of surgery, typically peaking during the first postoperative days, driven by the hepatic acute-phase response stimulated by IL-6 and TNF-α [38]. This postoperative hyperfibrinogenemia directly augments clot formation by increasing the fibrin network density and resistance to fibrinolysis, contributing to the net prothrombotic shift observable on viscoelastic testing as an increase in clot amplitude parameters (MA on TEG, MCF on ROTEM) [12,49]. Clinically, elevated preoperative fibrinogen has been independently associated with DVT risk in femoral and pelvic fracture patients, with an odds ratio of approximately 2.09 in multivariate analyses [50]. Lin et al. further demonstrated that while D-dimer has higher sensitivity for VTE detection (AUC 0.73), fibrinogen provides superior specificity (0.777), and their combination significantly improves the positive predictive value for VTE in patients older than 60 years [43]. As a reflection of clot structural properties, fibrinogen correlates directly with TEG/ROTEM amplitude parameters and represents a mechanistic link between the inflammatory response and viscoelastic hypercoagulability, making it uniquely suited to integrated biomarker–TEG/ROTEM assessment strategies [12].

### 4.4. Thrombin–Antithrombin (TAT) Complexes: A Direct Marker of Thrombin Generation

Thrombin–antithrombin (TAT) complexes are formed when thrombin—generated through coagulation cascade activation—is neutralized by its principal inhibitor, antithrombin III [51]. Unlike fibrinogen and D-dimer, which are downstream products of coagulation and fibrinolysis, respectively, TAT represents a direct marker of recent in vivo thrombin generation [15,51]. In orthopedic surgery patients, TAT levels undergo rapid and marked elevation from the first postoperative day, reflecting the intensity of tissue factor-driven thrombin burst triggered by surgical trauma [52]. Because TAT complexes are rapidly cleared from the circulation, they primarily reflect recent thrombin generation rather than sustained thrombin activity, making their interpretation highly dependent on the timing of sample collection [53,54]. A key prospective study by Lin et al. (2022) in 256 THA/TKA patients demonstrated that TAT measured on postoperative day 1 (POD-1) is a significant early predictor of DVT detected on POD-7 by Doppler ultrasonography, with a receiver operating characteristic curve (ROC) area under the curve (AUC) superior to that of D-dimer and plasmin–antiplasmin complex (PIC) at the same time point [52]. The authors proposed a nomogram incorporating TAT, surgical site, and leg swelling that achieved AUC values of 0.836 (development dataset) and 0.957 (validation dataset), substantially outperforming the Caprini risk score alone [55]. These findings position TAT as a particularly promising early biomarker for individualized thromboprophylaxis decisions, especially given that D-dimer is typically uninformative on POD-1 due to universal elevation. However, TAT is not yet widely implemented in routine clinical monitoring, primarily due to limited assay standardization and the absence of validated orthopedic-specific reference ranges and cutoff values across different patient populations and surgical procedures—an area explicitly identified as a priority for future research [52].

### 4.5. Prothrombin Fragments F1+2: Upstream Marker of Thrombin Generation

Prothrombin fragments 1+2 (F1+2) are released during the conversion of prothrombin to thrombin by the prothrombinase complex (FXa/FVa), and thus serve as an upstream, stoichiometric marker of thrombin generation [56]. With a plasma half-life of approximately 90 min—considerably longer than TAT—F1+2 provides a broader temporal window for detecting ongoing coagulation activation and is excreted in urine, where it remains detectably elevated for several days after major surgery [56]. In THA patients, plasma F1+2 is significantly elevated as early as the day of surgery and remains elevated through postoperative day 6, indicating sustained thrombin generation well beyond the operative window [56]. Urine F1+2 demonstrated a complementary but imperfectly correlated pattern, suggesting that the two compartments may capture different phases of coagulation activation. In combination with TAT, F1+2 provides a complementary picture of the thrombin generation axis: TAT reflects the rate of active thrombin formation and neutralization, while F1+2 reflects cumulative prothrombin activation over a longer timeframe [56]. Both markers have been proposed as components of integrated monitoring panels—alongside D-dimer and fibrinogen—that may better characterize the overall prothrombotic state than any single marker alone [51,52].

### 4.6. Plasminogen Activator Inhibitor-1 (PAI-1): The Fibrinolytic Brake

Plasminogen activator inhibitor-1 (PAI-1) is the principal endogenous inhibitor of fibrinolysis, acting by irreversibly binding and inactivating tissue plasminogen activator (t-PA) and urokinase, thereby preventing plasmin-mediated clot dissolution [57]. In the orthopedic perioperative context, PAI-1 levels are upregulated by surgical stress, elevated IL-6, and the acute-phase response, resulting in an early postoperative suppression of fibrinolysis that consolidates the prothrombotic state established by coagulation activation [38]. PAI-1 on postoperative day 1 has been associated with VTE occurrence in THA patients: in a prospective study of 170 patients, PAI-1 levels on POD-1 above a cutoff of 53.5 ng/mL yielded 100% sensitivity and 67% specificity for VTE prediction, with the combination of PAI-1 and soluble fibrin achieving a negative agreement rate of 98% [57]. Importantly, this predictive value was observed only in patients receiving mechanical prophylaxis and was attenuated when pharmacological anticoagulation (fondaparinux) was administered, suggesting that PAI-1 may be most clinically informative in settings where the fibrinolytic suppression is not pharmacologically offset [57].

### 4.7. Von Willebrand Factor (vWF): Endothelial Activation Marker

Von Willebrand factor (vWF)—a large multimeric glycoprotein released from endothelial Weibel-Palade bodies upon vascular injury or inflammatory stimulation—plays a critical role in primary hemostasis by mediating platelet adhesion to damaged subendothelium and acting as a carrier for Factor VIII [58]. In elective orthopedic surgery, perioperative vWF dynamics follow a characteristic biphasic pattern: an intraoperative decrease (attributed to consumptive processes) followed by a significant postoperative rise consistent with an acute-phase response, with Factor VIII levels reaching up to 4.5 IU/mL on POD-1 [58]. Elevated postoperative vWF and FVIII have been independently correlated with thrombotic risk, and FVIII levels exceeding 2.30 IU/L have been shown to be independently associated with VTE in population-based studies [58]. In orthopedic trauma, vWF also reflects the extent of endothelial activation and is therefore a functionally meaningful biomarker of the endothelial injury component of Virchow’s triad, complementing the coagulation activation markers discussed above.

### 4.8. Limitations of Conventional Tests and the Case for Multimarker Monitoring

A consistent finding across the studies reviewed is that no single biomarker provides sufficient sensitivity and specificity to serve as a standalone perioperative VTE predictor in orthopedic trauma patients [14,44,45]. PT, aPTT, and INR—the standard clinical battery—are systematically insensitive to hypercoagulable states [37]. D-dimer suffers from near-universal postoperative elevation. Fibrinogen lacks sensitivity. TAT and F1+2 lack standardized cutoffs. PAI-1 is context-dependent. vWF reflects only the endothelial component. This convergent evidence strongly supports the development of integrated multimarker monitoring panels—encompassing markers of thrombin generation (TAT, F1+2), fibrin formation (fibrinogen), fibrin degradation (D-dimer), and fibrinolytic suppression (PAI-1)—potentially complemented by viscoelastic hemostatic assays (TEG/ROTEM) capable of capturing the dynamic, whole-blood hemostatic phenotype. Such integrated approaches represent the conceptual and methodological frontier in perioperative VTE risk stratification in this patient population (Table 3).

## 5. Thromboelastography and Rotational Thromboelastometry—Principles, Parameters, and Perioperative Findings

### 5.1. Historical Context and Technological Principles

Thromboelastography (TEG) was first developed in 1948 by Helmut Hartert at the University of Heidelberg, with clinical application beginning in the 1960s in the context of liver transplantation surgery. Initially used to identify coagulopathy during surgery and guide blood product administration, studies of surgical patients have shown that TEG may decrease the use of blood products by up to 40%, as well as reduce postoperative length of stay and risk of reoperation [59]. Over the subsequent decades, viscoelastic hemostatic assays (VHAs) evolved from niche intraoperative monitoring tools into validated point-of-care systems with broad perioperative applications. The fundamental principle of TEG is the in vitro detection and quantification of dynamic changes in the viscoelastic properties of a blood sample during clotting under low shear stress. The system consists of two chambers simultaneously examining a blood sample in duplicate to reduce sampling and measurement errors. Each chamber contains a disposable cup where a blood sample is placed and a detection pin suspended at its center; the cup oscillates around the pin in a limited arc, and induced pin movement is recorded as a function of time [60]. This configuration allows TEG to capture the entire hemostatic process—from initial fibrin strand formation through clot consolidation and eventual fibrinolysis—in a single continuous graphical tracing. Rotational thromboelastometry (ROTEM) is a digitized modification of conventional TEG in which the cup remains stationary while the detection pin oscillates. Rapid TEG (r-TEG) uses tissue factor instead of kaolin-cephalin reagent to activate blood coagulation; because tissue factor triggers the extrinsic pathway, which involves fewer coagulation factors, the test can be completed within 15 min, making it particularly useful for managing massive transfusions in trauma patients [60]. Both platforms provide whole-blood assessment under near-physiological conditions, incorporating the contributions of cellular elements—erythrocytes, platelets, and leukocytes—that are absent from plasma-based conventional coagulation tests.

### 5.2. TEG Parameters: Definitions and Physiological Correlates

The TEG tracing generates five primary parameters with distinct physiological correlates. R time is the interval from the start of the assay to the point where clot strength reaches an amplitude of 2 mm; K is the time measured from R to when amplitude reaches 20 mm; the α-angle is generated from the x-axis and a line drawn from R to the point where K intersects 20 mm of amplitude; MA is the highest amplitude reached; and Ly30 is the percent decrease in amplitude 30 min after MA [61]. Each parameter reflects a distinct phase of hemostasis. R time largely reflects the adequacy of coagulation factors and is the most sensitive parameter to measure the effects of heparin therapy, including low-molecular-weight heparin (LMWH). A shortened R time of less than four minutes can indicate a procoagulant state requiring anticoagulation, whereas a prolonged R time beyond eight minutes may signify factor deficiency, hemodilution, or heparin effect [11]. K time and the α-angle together characterize the kinetics of fibrin formation and cross-linking: a decrease in K and a larger α-angle indicate faster clot coagulation, consistent with a prothrombotic phenotype [62]. MA is the maximum clot amplitude reached during the assay and reflects the ultimate mechanical strength of the platelet–fibrin clot. It is determined primarily by platelet number and function together with fibrinogen-mediated platelet interactions, whereas other coagulation factors play a smaller role. Although elevated MA is commonly associated with hypercoagulability, it should not be interpreted as a direct surrogate of thrombin generation, as it reflects clot strength rather than thrombin enzymatic activity [63,64]. Ly30 is the percent decrease in clot strength 30 min after achieving maximum amplitude, and increased Ly30 indicates hyperfibrinolysis [59]. In addition to these five core parameters, the TEG system provides a composite coagulation index (CI), calculated as: CI = −0.2454R + 0.0184K + 0.1655MA − 0.0241α − 5.0220. Normal values lie within −3.0 and +3.0. A hypercoagulable state is defined as CI greater than +3.0, and coagulopathy as CI less than −3.0 [60]. Major orthopedic surgery leads to massive tissue factor release and substantial triggering of coagulation, and the CI was investigated as a point-of-care index to reflect the antithrombotic effect of LMWH in patients undergoing total knee arthroplasty, with positive CI values beyond +3.0 indicating hypercoagulability [65]. The hypercoagulable TEG profile is therefore characterized by a constellation of findings: shortened R time, shortened K time, elevated α-angle, elevated MA, and decreased Ly30—the inverse pattern of what is observed in hemorrhagic coagulopathy [59].

### 5.3. ROTEM Parameters: Nomenclature, Assay Variants, and Reference Ranges

TROTEM employs a parallel parameter nomenclature. The principal correspondences are: clotting time (CT) ≈ R time; clot formation time (CFT) ≈ K time; α-angle (shared); maximum clot firmness (MCF) ≈ MA; and lysis index (LI30) ≈ Ly30. The results from TEG and ROTEM cannot be directly interchanged, and manufacturers for each product have specific normal ranges. The alpha angle retains its name across both platforms, though its geometric derivation differs slightly between systems [59]. The prevalent view is that the two tests are equivalent, with interchangeable clinical interpretations; however, comparative studies suggest that absolute values show significant differences for most measurements between platforms, and this distinction becomes relevant when cross-study comparisons are attempted [10]. ROTEM offers distinct assay variants activating different coagulation pathways, enabling differential diagnosis of hemostatic defects. EXTEM activates the extrinsic (tissue factor) pathway; INTEM activates the intrinsic (contact) pathway; FIBTEM adds a platelet inhibitor (cytochalasin D) to isolate the fibrin contribution to clot firmness; APTEM adds aprotinin to confirm hyperfibrinolysis; and HEPTEM incorporates heparinase to neutralize heparin effects and unmask underlying coagulation status. The diagnostic performance of ROTEM is enhanced by combining assays—specifically EXTEM with FIBTEM, EXTEM with APTEM, or INTEM with HEPTEM—as each combination targets a different mechanistic component of the hemostatic process [66,67,68]. Validated multicenter reference ranges for ROTEM have been established. Reference values include: INTEM CT 137–246 s, CFT 40–100 s, MCF 52–72 mm; EXTEM CT 42–74 s, CFT 46–148 s, MCF 49–71 mm; FIBTEM MCF 9–25 mm. These ranges showed consistent reproducibility across centers, with coefficients of variation of 1–5% for clot firmness and α-angle, and 3–12% for CT and CFT [69]. Values falling below the lower limit for CT or CFT, or exceeding the upper limit for MCF, indicate a hypercoagulable profile within the respective pathway assessed.

### 5.4. TEG and ROTEM Findings in Orthopedic Surgery: Evidence of Perioperative Hypercoagulability

The application of VHAs to orthopedic surgery has produced consistent evidence of perioperative hypercoagulability that is systematically undetected by conventional coagulation tests. A landmark systematic review and meta-analysis by Brown et al. examined the capacity of TEG and ROTEM to detect hypercoagulability across perioperative settings [12]. Of the 35 included studies encompassing 8939 patients with 717 thrombotic events, elevated MA was a statistically significant indicator of hypercoagulability across at least one perioperative time point in 17 studies (50%), representing 6348 patients (72%). The average MA value used to define hypercoagulability was above 66.70 mm. Using a prepublished threshold of 65 mm, the combined effect of MA on postoperative VTE development yielded an odds ratio of 1.31 (95% CI 0.74–2.34), with 46% sensitivity and 62% specificity. Critically, when hypercoagulability was assessed across the perioperative period, TEG consistently demonstrated a hypercoagulable profile beginning on postoperative day 1 (POD-1) [12]. In studies focused specifically on orthopedic trauma patients, ROTEM has demonstrated clear associations between hypercoagulable patterns and VTE outcomes. Tsantes et al. conducted a prospective case–control study in patients undergoing hip fracture surgery and found that fractured patients showed significantly lower INTEM and EXTEM CT values (*p* = 0.008 and *p* = 0.012, respectively) and significantly lower INTEM and EXTEM CFT values (*p* < 0.001) compared to the controls [70]. These differences were further amplified postoperatively, with INTEM MCF, A10, and α-angle all increasing significantly after surgery, indicating that the surgical procedure itself further augments the pre-existing coagulation potential. Importantly, this higher coagulation activity was detectable by ROTEM shortly after injury, even when undetectable by conventional coagulation assays [70]. In a subsequent study by the same group focused on symptomatic VTE prediction after hip fracture surgery, ROTEM performance was promising in terms of its association with symptomatic VTE, with preoperative INTEM and EXTEM CFT and postoperative INTEM MCF demonstrating meaningful diagnostic accuracy for clinically significant thromboembolic events. The authors noted, however, that until ROTEM performance is validated in studies applying a diagnostic gold standard in all patients undergoing parallel ROTEM testing, these tools should be used with caution as a standalone clinical screening instrument [13]. A 2026 prospective study in elderly patients with femoral fractures further refined the temporal dynamics of ROTEM-detected hypercoagulability. DVT patients showed significantly increased EXTEM maximum clot firmness (67.6 ± 1.7 vs. 62.2 ± 5.7 mm; *p* = 0.024) and shorter clot formation times (92.0 ± 9.3 vs. 116.1 ± 32.0 s; *p* = 0.041) compared to non-DVT patients [71]. An EXTEM-MCF threshold of ≥66 mm at postoperative day 3 demonstrated both high sensitivity and specificity for DVT prediction. Furthermore, temporal monitoring was identified as potentially superior to single-timepoint assessments: DVT patients showed progressive fibrinogen increases of +43.7 mg/dL/day, compared to reductions of −9.5 mg/dL/day in non-DVT patients over the postoperative period [71]. For elective joint arthroplasty, Tsantes et al. additionally demonstrated the prognostic utility of ROTEM for hemorrhagic risk stratification. In hip fracture surgery, a preoperative FIBTEM MCF ≤ 11 mm and postoperative FIBTEM MCF ≤ 15 mm were associated with markedly elevated risks of excessive bleeding, with odds ratios of 44.8 and 23.0, respectively, enabling preoperative blood-sparing strategies in high-risk patients [72]. These findings illustrate the bidirectional utility of VHAs in orthopedic trauma—capable of identifying both pro-thrombotic and pro-hemorrhagic hemostatic states within the same patient population.

### 5.5. TEG/ROTEM vs. Conventional Coagulation Tests: Comparative Diagnostic Performance

A fundamental advantage of VHAs over conventional coagulation tests lies in their capacity to assess whole-blood viscoelastic dynamics rather than isolated pathway activation times. Conventional tests such as PT, aPTT, INR, and ACT provide only static evaluation and are not designed for the assessment of dynamically changing coagulation conditions during the perioperative period, whereas TEG and ROTEM produce a rapid numerical and graphical representation, enabling real-time detection and targeted hemostatic therapy [11]. This superiority has been quantified in orthopedic-specific analyses. In one orthopedic clinical experiment, the AUC values for TEG α-angle, MA, and CI were 0.665, 0.669, and 0.808, respectively, demonstrating that the diagnostic ability of these parameters is superior to that of INR and PT in detecting postoperative hypercoagulability in both knee and hip surgery groups [62]. The mechanistic basis for this advantage relates to the multi-phase assessment offered by VHAs: while conventional tests such as PT and INR reflect only the time to fibrin formation, they do not capture clot formation kinetics, maximum clot firmness, or fibrinolysis—precisely the phases most relevant to thrombotic risk stratification [66]. Similarly, standard clotting tests were not different between patients with and without thromboembolic complications in major non-cardiac surgery, whereas ROTEM parameters—specifically INTEM and EXTEM A10, CFT, and MCF—were [66].

### 5.6. Limitations of TEG and ROTEM in the Orthopedic Context

Despite their advantages, VHAs carry important limitations that must be acknowledged in the orthopedic perioperative setting. First, results are sensitive to preanalytical variables including sample processing time, temperature, and reagent selection; values may fluctuate based on the manufacturer and reagent used, and adequate interpretation requires that normal limits for the specific platform in use be established at each institution [59]. Second, the absence of endothelial contribution is a fundamental physiological limitation, as TEG and ROTEM do not account for endothelial–blood interactions that modulate in vivo thrombotic risk [63]. Furthermore, although TEG and ROTEM provide a global assessment of clot formation, they cannot differentiate the molecular pathways responsible for coagulation activation. Distinct fracture patterns and orthopedic procedures may preferentially activate tissue factor- or contact pathway-mediated thrombin generation, yet these mechanisms frequently produce similar viscoelastic profiles. Therefore, VHAs should be interpreted as functional assays of overall clot dynamics rather than pathway-specific tests. Third, while the evidence for VHAs in trauma and major surgery is growing, the orthopedic-specific literature remains limited by small sample sizes, heterogeneous patient populations, and the absence of randomized controlled trials with clinical outcome endpoints [12]. Fourth, standardized hypercoagulable thresholds for orthopedic-specific populations remain undefined: the variability in MA cutoffs used across studies—ranging from 65 mm to over 70 mm—limits direct comparisons across studies and the generalizability of individual institution findings [12].

## 6. Correlations Between Coagulation Biomarkers and Viscoelastic Testing—Complementary Assessment of Perioperative Hypercoagulability

### 6.1. Conceptual Framework: Two Complementary Levels of Hemostatic Analysis

The assessment of hypercoagulability in orthopedic trauma surgery operates at two distinct but deeply interconnected analytical levels. Plasma-based coagulation biomarkers—D-dimer, fibrinogen, TAT complexes, F1+2, PAI-1, and vWF—capture molecular events along specific mechanistic axes: thrombin generation, fibrin substrate availability, fibrinolytic suppression, and endothelial activation. Viscoelastic hemostatic assays—TEG and ROTEM—measure the functional aggregate of these molecular processes as expressed in the mechanical behavior of whole blood during clot formation and dissolution. Together, these two levels of analysis are not competitive, but complementary: biomarkers provide mechanistic resolution and temporally precise molecular signaling, while VHAs provide integrative functional assessment of the net hemostatic phenotype and real-time point-of-care data suitable for therapeutic decision-making. This complementarity is physiologically grounded. No single biomarker or viscoelastic parameter captures the complete hemostatic process in isolation. D-dimer reflects downstream fibrinolytic activity but not the dynamics of active clot formation; fibrinogen concentration predicts clot substrate availability, but not the efficiency of platelet–fibrin interaction at the moment of clot consolidation. MA and MCF capture the final biomechanical properties of the clot rather than the individual molecular pathways responsible for its formation; therefore, they cannot distinguish the relative contribution of platelet activation, fibrinogen, thrombin generation, or fibrinolytic inhibition to the hypercoagulable phenotype. The emerging consensus in the orthopedic trauma literature is that multimodal monitoring combining plasma biomarkers with VHAs yields superior risk stratification compared to either approach applied in isolation [62,73].

### 6.2. Fibrinogen and TEG/ROTEM Clot Strength Parameters

Among all biomarker–VHA correlations, the relationship between plasma fibrinogen concentration and TEG/ROTEM clot strength parameters is the most extensively studied and mechanistically direct. Fibrinogen serves as the primary substrate for thrombin-mediated clot formation, and its functional contribution to clot mechanical properties is independently reflected in MA/MCF (composite platelet–fibrin strength) and in the fibrinogen-specific FIBTEM-MCF parameter of ROTEM, which isolates the fibrin contribution by blocking platelet activation with cytochalasin D. The correlation between plasma fibrinogen and viscoelastic clot strength has been documented across multiple perioperative settings. In a study of bariatric surgery patients—a population with VTE risk profiles overlapping significantly with orthopedic trauma patients—a direct correlation was found between baseline fibrinogen levels and TEG MA (R = 0.431, *p* = 0.001) and clot elasticity G (R = 0.387, *p* = 0.003), with ROC curve analysis showing that fibrinogen levels can predict clot strength G ≥ 11 dynes/cm^2^ with AUC = 0.680 [74]. Fibrinogen levels were significantly higher in patients with MA ≥ 68 mm compared to those with MA < 68 mm (*p* = 0.025), underscoring that elevated fibrinogen translates directly into measurable increases in viscoelastic clot strength [74]. In adult spinal deformity surgery—another major orthopedic procedure—TEG variables G, K, and α-angle all correlated significantly with preoperative fibrinogen concentration (*p* < 0.05), and total blood loss correlated inversely with fibrinogen (r = −0.51, *p* < 0.05), confirming the dual role of fibrinogen as both a hypercoagulable driver and a hemorrhagic risk modifier [75]. In the context of total joint arthroplasty specifically, Wanderling et al. demonstrated that patients undergoing TJA exhibit preoperative elevations of fibrinogen and microparticle tissue factor (MP-TF) compared to healthy controls, representing a pre-existing hemostatic dysregulation that precedes the surgical insult [48]. This finding has direct implications for interpreting perioperative VHA results: the elevated MA or MCF observed postoperatively in THA/TKA patients may reflect both a surgery-driven increment and a pre-existing hypercoagulable substrate detectable already at the baseline. Importantly, however, the fibrinogen–FIBTEM correlation reveals a mechanistic dissociation relevant to DVT pathogenesis. In the 2026 prospective femoral fracture study by Gianesello et al., plasma fibrinogen levels diverged progressively between DVT and non-DVT patients over the postoperative period, yet FIBTEM-MCF did not differ significantly between the two groups [71]. This apparent discrepancy is mechanistically explained by the fact that the Clauss fibrinogen assay measures plasma fibrinogen concentration as a substrate, while FIBTEM-MCF reflects the functional contribution of fibrin polymerization to clot firmness—two distinct but related properties. The finding suggests that in this population, DVT development was driven primarily by platelet-mediated clot amplification rather than fibrin-dependent mechanisms, a distinction that only the combined use of FIBTEM alongside standard EXTEM/INTEM can reveal [71].

### 6.3. Thrombin Generation Markers and Viscoelastic Clot Initiation Parameters

The relationship between thrombin generation biomarkers—principally TAT complexes and F1+2 fragments—and the clot initiation parameters of TEG/ROTEM (R time and CT) provides a mechanistically coherent axis of correlation. TAT complexes reflect recent thrombin generation, whereas F1+2 fragments provide a more integrated estimate of cumulative prothrombin activation over time. Both biomarkers are mechanistically linked to clot initiation parameters (R time and CT), although they provide complementary temporal information [53,54]. Principal component analysis of TEG parameters in trauma patients has confirmed that the time to maximum rate of thrombin generation (TMRTG) correlates with TAT levels, indicating that viscoelastic assessment of clot initiation kinetics is biologically reflective of the thrombin generation axis [76]. Elevated TAT complexes measured on POD-1 after THA and TKA are predictive of subsequent DVT, with reported AUC values ranging from 0.836 to 0.957 [52,55]. Biologically, this thrombin-generation signal is consistent with the shortened R time/CT and increased α-angle commonly observed on perioperative TEG/ROTEM assessments of hypercoagulability [62]. The two measurement modalities, therefore, converge on the same biological signal—accelerated thrombin generation driving accelerated clot formation—from complementary analytical perspectives: TAT quantifies the molecular intensity of thrombin–antithrombin neutralization, while R time and CT capture its functional consequence in whole blood dynamics. Combining both provides both mechanistic confirmation and functional validation of the hypercoagulable state. F1+2 fragments, as markers of cumulative prothrombin conversion activity, extend this relationship over time. Given the documented sustained elevation of plasma F1+2 through POD-6 in THA patients, and the parallel observation that TEG consistently demonstrates a procoagulant profile beginning on POD-1 and persisting through the first postoperative week [56], these two approaches provide convergent evidence of a prolonged hypercoagulable window that extends well beyond the operative period itself. Serial measurement of both F1+2 and TEG CI over this window would provide a more complete picture of the temporal evolution of the prothrombotic state than either modality alone.

### 6.4. PAI-1, Fibrinolytic Suppression, and Ly30/LI30 Discordance

The correlation between PAI-1—the principal endogenous inhibitor of fibrinolysis—and the fibrinolysis parameters of TEG and ROTEM (Ly30 and LI30) represents one of the most clinically important, yet most frequently misinterpreted, relationships in the multimodal assessment of orthopedic hypercoagulability. Orthopedic surgery is characterized by a paradoxical hemostatic phenotype: while thrombin generation is markedly amplified (reflected in elevated TAT, shortened R time, elevated MA), fibrinolysis is simultaneously suppressed rather than activated—a state of hypofibrinolysis driven by PAI-1 upregulation in response to surgical stress, cytokine release, and tissue factor pathway activation [38,57]. This hypofibrinolytic phenotype is directly reflected in the near-zero Ly30 values and persistently elevated LI30 values characteristically observed in orthopedic patients postoperatively. In THA and TKA patients, Ly30 values remained within normal range but were consistently elevated relative to the preoperative baseline (*p* < 0.05), indicating a progressive trend toward hypofibrinolysis rather than hyperfibrinolysis throughout the perioperative period [77]. This is the inverse of the pattern observed in trauma-induced coagulopathy with massive hemorrhage, where Ly30 elevation is a marker of consumptive hyperfibrinolysis and carries major prognostic implications for mortality. The clinical significance of this dissociation is substantial. TEG and ROTEM are relatively insensitive to the moderate degrees of fibrinolytic suppression that characterize orthopedic hypercoagulability: because Ly30 values remain within the conventional normal range (0–8%) in most orthopedic patients, the viscoelastic assay fails to flag the degree of fibrinolytic suppression that is mechanistically driving VTE risk. Plasma PAI-1 measurement on POD-1 fills this gap precisely, providing a quantitative index of fibrinolytic inhibition that the VHA cannot reliably detect. The combined profile of elevated MA (VHA-detected hypercoagulability) with elevated PAI-1 > 53.5 ng/mL (biomarker-detected fibrinolytic suppression) therefore represents a more complete characterization of the dual-axis thrombotic risk—hypercoagulation plus hypofibrinolysis—than either parameter provides independently [57,77]. Furthermore, the study by Hasegawa et al. demonstrated that VTE onset after THA may be attributable to either a prothrombotic phenotype, a suppressed fibrinolytic state, or both simultaneously [57]. This heterogeneity in thrombotic mechanism—detectable by the combined SF/PAI-1 biomarker approach but not by VHA alone—underscores the importance of multimodal assessment in identifying high-risk patients for whom standard prophylaxis may be insufficient.

### 6.5. D-Dimer and Its Relationship to Postoperative VHA Dynamics

D-dimer occupies a unique position in the biomarker–VHA correlation framework because it is a downstream product of fibrinolytic clot dissolution, and therefore reflects a phase of hemostasis—the post-lysis degradation of cross-linked fibrin—that occurs temporally after the events captured by TEG and ROTEM. This temporal offset has direct clinical implications for the interpretation of concurrent D-dimer and VHA measurements in the orthopedic postoperative period. In THA and TKA patients, D-dimer and fibrin degradation products (FDPs) demonstrate a characteristic bimodal postoperative kinetic: an initial peak at 6 h postoperatively driven by intraoperative fibrinolysis, followed by a decline and subsequent rebound at 48 h reflecting ongoing fibrin turnover during the early thrombotic period [78]. This bimodal pattern is not captured by TEG/ROTEM, which measure clot formation at a fixed point in time rather than the cumulative fibrinolytic output over hours to days. The discordance between a hypercoagulable TEG profile (elevated MA, shortened R time, elevated CI) and simultaneously elevated D-dimer therefore does not represent a contradiction: the two findings reflect different phases of the coagulation–fibrinolysis cycle occurring in temporal sequence within the same patient [78]. The clinical implication is that D-dimer elevation at POD-1 or POD-7 cannot be interpreted in isolation without reference to the concurrent VHA profile and the temporal kinetics of the surgical procedure. D-dimer levels significantly correlated with total blood loss at POD-3 in arthroplasty patients (*p* = 0.007–0.044), suggesting that elevated early D-dimer reflects the degree of surgical fibrinolytic activation rather than necessarily indicating de novo VTE at that timepoint [78]. Combined monitoring—VHA at POD-1 to establish the functional coagulation phenotype, followed by D-dimer kinetics through POD-7—provides a more coherent clinical narrative than either measurement in isolation.

### 6.6. vWF, Endothelial Activation, and TEG Clot Initiation

The relationship between vWF—a marker of endothelial activation—and TEG/ROTEM parameters connects the endothelial injury axis of Virchow’s triad to the functional coagulation profile measured by VHAs. vWF stabilizes circulating factor VIII (FVIII) and mediates platelet adhesion to subendothelial collagen at sites of vascular injury; its postoperative elevation to levels of up to 4.5 IU/mL therefore directly contributes to both platelet-driven clot strength (MA/MCF elevation) and Factor VIII-driven amplification of thrombin generation (R time shortening, α-angle increase). The strong correlation between FVIII levels and all ROTEM parameters except CT—including MCF, CFT, and α-angle in both EXTEM and INTEM assays—confirms that the endothelial activation axis, measured biochemically through vWF/FVIII, is functionally expressed in the viscoelastic clot profile [58,79]. This mechanistic link is clinically relevant because vWF elevation identifies a specific endothelial-driven component of the hypercoagulable state that is separate from the fibrinogen-driven and platelet-driven components already discussed. A patient with elevated vWF/FVIII, elevated fibrinogen, and a procoagulant TEG profile represents a convergence of three independent prothrombotic axes—endothelial, substrate, and kinetic—that collectively confer a substantially higher VTE risk than any single marker would suggest.

### 6.7. Toward Multimodal Risk Stratification: Integration in Clinical Practice

The correlational evidence reviewed in this section supports a model of perioperative hypercoagulability assessment in which plasma biomarkers and VHAs are used in a defined complementary sequence rather than interchangeably. TEG and ROTEM provide rapid, point-of-care, whole-blood functional characterization of the hemostatic phenotype at a given moment—identifying whether the patient is in a hypercoagulable, normocoagulable, or hypocoagulable state and quantifying the degree of clot strength amplification and fibrinolytic suppression. Plasma biomarkers provide mechanistic resolution—identifying which specific axis is driving the phenotype (thrombin generation excess via TAT/F1+2; fibrin substrate amplification via fibrinogen; fibrinolytic shutdown via PAI-1; endothelial activation via vWF)—and enable longitudinal monitoring of the molecular trajectory over the postoperative period. Among the TEG parameters, K, α-angle, and MA demonstrate the most stable and consistent correlation with conventional coagulation biomarkers and are therefore the recommended reference indicators for diagnosing the hypercoagulable state in orthopedic patients [62]. Among the plasma biomarkers, TAT on POD-1 provides the earliest and most mechanistically specific signal of thrombin generation intensity [52]. The combination of a procoagulant VHA profile (MA > 66.7 mm or CI > +3.0) with concurrent elevation of TAT beyond the normal range (>0.55 mg/L FEU) and PAI-1 > 53.5 ng/mL may represent a convergent multimodal signature of high thrombotic risk in the orthopedic perioperative setting—a composite that identifies patients who may benefit from intensified or prolonged thromboprophylaxis beyond the standard protocol. Future prospective studies in orthopedic trauma populations are needed to validate such composite scoring approaches and establish evidence-based thresholds for therapeutic escalation.

## 7. Clinical Implications and Thromboprophylaxis Strategies—Toward Individualized Perioperative Management

### 7.1. From Population-Based to Individualized Thromboprophylaxis

For decades, thromboprophylaxis in orthopedic surgery was administered on a population basis—the same agent, dose, and duration prescribed to all patients undergoing a given procedure, regardless of individual hemostatic profile. This approach, while pragmatically efficient, fails to account for the wide interindividual variability in thrombotic risk that is now demonstrably measurable through the biomarker and viscoelastic tools reviewed in this manuscript. More recently, orthopedic prophylaxis has started to become more nuanced and individualized: modern trials focus on symptomatic VTE as outcomes, and there is a slow but accelerating move toward studying and recommending thromboprophylaxis based on individual patient attributes, in whom risk stratification and weighing of benefit versus hemorrhagic risk is becoming central to clinical decision-making [80,81]. This shift is not merely conceptual. Personalized approaches to VTE risk prediction are increasingly appealing in orthopedic surgery, but their implementation is hampered by the absence of standardized, validated risk assessment models specific to orthopedic populations, and no validated risk assessment models for bleeding have been established until now for evaluating hemorrhagic risk in this patient group [19,81,82]. The biomarkers and VHAs reviewed in this manuscript offer a pathway toward filling this gap, providing objective, quantitative hemostatic data that can stratify patients into distinct risk categories and inform therapeutic decisions with a precision that clinical risk scores alone cannot achieve.

### 7.2. Current Pharmacological Prophylaxis: Guidelines and Evidence Base

The major international guidelines provide a framework for pharmacological thromboprophylaxis in orthopedic trauma surgery. Current ACCP guidelines recommend the use of LMWH, low-dose unfractionated heparin, vitamin K antagonists, fondaparinux, apixaban, dabigatran, rivaroxaban, aspirin, or intermittent pneumatic compression devices for at least 10 to 14 days and up to 35 days in patients undergoing total hip or knee replacement; LMWH is suggested in preference to other pharmacological agents [19,82]. For hip fracture surgery specifically, LMWH remains the preferred first-line agent given the higher severity of trauma-induced coagulopathy and the older, more comorbid patient population. Direct oral anticoagulants (DOACs) have progressively assumed a central role in perioperative prophylaxis. Apixaban and rivaroxaban directly inhibit factor Xa, whereas dabigatran is a direct thrombin inhibitor; they have rapid onset, shorter half-lives allowing hemostasis recovery within two to three days, no dietary interactions affecting efficacy, and fewer drug interactions than vitamin K antagonists [82]. However, re-initiation timing requires careful individualization: re-initiation of dabigatran is recommended one to four hours post-surgery; for rivaroxaban, six to ten hours post-surgery; and for apixaban, 12 to 24 h post-surgery—the wider initiation window for apixaban providing more time for establishing hemostasis [80]. A significant ongoing controversy in the orthopedic prophylaxis literature concerns the role of aspirin. At the 2022 International Consensus Meeting on Venous Thromboembolism, delegates recommended low-dose aspirin as an appropriate option for routine VTE prophylaxis following primary total joint arthroplasty [83]. However, evidence remains conflicting. The CRISTAL randomized trial demonstrated a significantly lower incidence of symptomatic VTE with enoxaparin compared with aspirin, suggesting that aspirin may be inadequate for some patients, particularly those at elevated thrombotic risk. This discrepancy highlights the need for improved risk stratification strategies capable of identifying patients who may benefit from more potent anticoagulant prophylaxis [84].

### 7.3. Mechanical Prophylaxis: Role and Integration

Pharmacological prophylaxis is not deployed in isolation. During hospitalization, dual prophylaxis with an intermittent pneumatic compression device for at least 18 h daily, combined with an antithrombotic agent, is recommended; in patients at increased risk for bleeding, a pneumatic compression device alone or no prophylaxis is favored over pharmacological prophylaxis [19]. Graduated compression stockings and early postoperative mobilization represent additional components of the mechanical prophylaxis strategy, with particular relevance in the immediate postoperative window when pharmacological anticoagulation is withheld to permit wound hemostasis. The hemostatic rationale for mechanical prophylaxis is directly linked to the venous stasis component of Virchow’s triad described in Section 3: intermittent pneumatic compression devices counteract the stasis-driven endothelial activation and fibrinolytic suppression that characterize the early postoperative period, and their benefit is additive to pharmacological anticoagulation rather than redundant. In patients with VHA-documented hypercoagulability but elevated hemorrhagic risk—for instance, a patient with MA > 67 mm but preoperative FIBTEM-MCF ≤ 11 mm—dual mechanical prophylaxis may represent the safest initial strategy pending achievement of adequate surgical hemostasis.

### 7.4. TEG/ROTEM-Guided Anticoagulation: Evidence and Potential

The concept of VHA-guided anticoagulation in orthopedic surgery—using TEG or ROTEM findings to individualize the choice, timing, and dosing of thromboprophylaxis—is supported by growing, though not yet definitive, evidence. The R time and CI of TEG can sensitively detect the anticoagulant effect of LMWH in blood, and R has a dependent relationship with the peak serum concentration of LMWH, suggesting a potential correlation between R time and LMWH-specific dose and frequency [65]. Unlike anti-Xa assays, which quantify circulating LMWH activity, TEG and ROTEM assess the net functional effect of anticoagulation on whole-blood coagulation dynamics. Therefore, VHAs may provide complementary information beyond plasma drug concentration measurements. In the context of extremity trauma, a secondary analysis of the PREVENT CLOT trial demonstrated that admission R time ≥ 2.0 was independently associated with a 2.1-fold increased odds of postoperative VTE (OR 2.13; 95% CI 1.06–4.28; *p* < 0.001), even though MA thresholds of ≥65, ≥69, or ≥72 mm showed no significant association with VTE in that population [85]. This finding challenges the predominant focus on MA as the sole VHA hypercoagulability marker and suggests that the clot initiation axis—reflected in R time and CT—may carry equal or greater predictive weight in certain orthopedic trauma contexts. Although the EAST guideline addresses bleeding patients rather than thromboprophylaxis, its findings support the broader principle that VHA-guided hemostatic management may provide clinically relevant information beyond conventional coagulation tests. Whether this paradigm can be extended to individualized anticoagulant strategies in orthopedic trauma remains to be established in prospective studies [86].

### 7.5. Biomarker-Informed Risk Stratification: A Proposed Framework

Synthesizing the evidence reviewed in this manuscript, a practical multimodal monitoring framework can be proposed for the perioperative assessment of hypercoagulability in orthopedic trauma surgery (Table 4). This framework operates across three temporal windows, each aligned with the biomarker and VHA evidence reviewed in Section 4, Section 5 and Section 6. In the preoperative window, baseline assessment should include plasma fibrinogen, D-dimer with age-adjusted cutoff, platelet count, and—where available—a preoperative ROTEM or TEG to establish the patient’s hemostatic phenotype. Preoperative FIBTEM-MCF provides a hemorrhagic risk stratification index, while preoperative EXTEM/INTEM CT and MA establish the baseline coagulable state against which postoperative changes will be measured. Preoperative fibrinogen > 3.543 g/L, platelet count ≥ 277 × 10^9^/L, or a hypercoagulable VHA profile should prompt enhanced pharmacological prophylaxis rather than aspirin monotherapy [43,71]. In the early postoperative window (POD-1), the most predictively powerful measurement timepoint identified across the literature, TAT complexes should be measured as the primary early marker of thrombin generation intensity. PAI-1 > 53.5 ng/mL provides a fibrinolytic suppression index independent of VHA [57]. Concurrent TEG/ROTEM at POD-1 establishes the functional hemostatic phenotype: a CI > +3.0 or MA > 66.7 mm in combination with elevated TAT and PAI-1 constitutes a convergent high-risk signature justifying early DOAC or LMWH initiation and consideration of extended duration prophylaxis. In the monitoring window (POD-3 through POD-7), serial D-dimer kinetics—specifically the direction of change rather than absolute value—provide the most accessible clinical signal. A progressively rising D-dimer trajectory through POD-7 in a patient on standard prophylaxis suggests inadequate anticoagulant effect and warrants reassessment of agent and dose. EXTEM-MCF ≥ 66 mm at POD-3, as documented in the femoral fracture literature, correlates with DVT on systematic screening at POD-5 [71].

### 7.6. Limitations and Future Directions

Several important limitations constrain the immediate clinical implementation of the multimodal framework proposed above. First, no prospective randomized trial has yet demonstrated that VHA-guided or biomarker-guided thromboprophylaxis individualization reduces symptomatic VTE rates compared to standard protocol-driven care in orthopedic trauma patients. The evidence base is predominantly observational and retrospective, with heterogeneous patient populations and inconsistent VHA threshold definitions across studies. Second, the cost-effectiveness of adding TEG, ROTEM, TAT, and PAI-1 to routine perioperative monitoring has not been formally evaluated in an orthopedic trauma context. Third, logistical constraints—availability of VHA platforms at point of care, assay turnaround times, and laboratory expertise—limit implementation in many center-level settings, particularly in emergency trauma contexts. Future research priorities include prospective validation of composite risk scores incorporating both biomarkers and VHA parameters in large orthopedic trauma cohorts; randomized controlled trials comparing standard versus biomarker/VHA-guided thromboprophylaxis strategies; development of automated alert systems for convergent high-risk hemostatic profiles; and pharmacokinetic–pharmacodynamic studies correlating LMWH and DOAC plasma levels with real-time TEG/ROTEM parameters to enable truly personalized anticoagulant dosing.

## 8. Conclusions and Future Perspectives

### 8.1. Summary of Principal Findings

This narrative review examined the pathophysiological mechanisms, clinical evidence, and practical implications of hypercoagulability assessment in orthopedic trauma surgery, with a focus on two complementary analytical modalities: plasma-based coagulation biomarkers and viscoelastic hemostatic assays. The evidence synthesized across Section 3, Section 4, Section 5, Section 6 and Section 7 supports a coherent and clinically actionable picture of perioperative hemostatic dysregulation in this patient population. Orthopedic trauma surgery generates a predictable and temporally well-characterized prothrombotic phenotype driven by the convergence of all three components of Virchow’s triad: venous stasis imposed by operative positioning, tourniquet application, and postoperative immobility; endothelial injury from surgical dissection, bone manipulation, and implant placement; and a systemic hypercoagulable shift driven by thrombin generation amplification, acute-phase fibrinogen elevation, fibrinolytic suppression mediated by PAI-1, and vWF-driven platelet activation. This state is not a uniform phenomenon: it exhibits significant interindividual variability in onset, intensity, and duration—variability that standard clinical risk scores and conventional coagulation tests are insufficient to capture. Among plasma biomarkers, TAT complexes emerge as the earliest and most mechanistically specific marker of thrombin generation excess, demonstrating diagnostic AUC values of 0.836 to 0.957 for DVT prediction at POD-1 in hip fracture patients—a predictive performance substantially exceeding D-dimer alone. Fibrinogen provides both a substrate-level predictor of clot strength and a bidirectional hemostatic risk index, with low preoperative levels predicting hemorrhagic complications and elevated postoperative levels predicting thrombotic events. PAI-1, though less routinely measured, captures the fibrinolytic suppression axis that neither conventional coagulation tests nor standard VHA parameters adequately reflect. D-dimer, despite its well-recognized specificity limitations, retains clinical utility as a longitudinal monitoring tool when interpreted through its temporal kinetic trajectory rather than a single absolute value. vWF connects the endothelial activation axis directly to platelet-mediated clot amplification, particularly relevant in arthroplasty and hip fracture patients with perioperative catecholamine and inflammatory surges. Viscoelastic hemostatic assays—TEG and ROTEM—add the functional dimension that no plasma biomarker panel can provide: real-time, whole-blood assessment of clot formation kinetics, clot strength, and fibrinolysis in a single integrated measurement. The consistent demonstration of hypercoagulable TEG/ROTEM profiles beginning on POD-1 across multiple orthopedic procedure types, the superior diagnostic performance of CI and MA over INR and PT for postoperative hypercoagulability, and the prospectively validated predictive value of EXTEM-MCF ≥ 66 mm at POD-3 for DVT constitute a compelling argument for the incorporation of VHAs into the perioperative monitoring protocol for high-risk orthopedic patients. The bidirectional utility of VHAs—identifying both pro-thrombotic states (elevated MA/MCF, shortened CT/R) and pro-hemorrhagic states (low FIBTEM-MCF, prolonged CT/R)—provides a risk stratification framework that simultaneously addresses both ends of the hemostatic spectrum. The correlational evidence reviewed in Section 6 establishes that biomarkers and VHAs are not competing but complementary modalities. Fibrinogen and TEG clot strength parameters (MA, K, α-angle) correlate consistently and reflect the same underlying substrate-driven mechanism, while PAI-1 captures fibrinolytic suppression that Ly30/LI30 routinely misses, and D-dimer kinetics provide temporal information that single-timepoint VHAs cannot. Together, these modalities form a multimodal hemostatic monitoring system capable of characterizing the full dimensionality of perioperative hypercoagulability in orthopedic trauma surgery.

### 8.2. Clinical Implications

The principal clinical implication of this review is that the binary, procedure-determined approach to thromboprophylaxis in orthopedic surgery—the same drug and duration for all patients undergoing a given operation—is biologically inadequate and methodologically obsolete in light of the available hemostatic monitoring evidence. The persistent VTE rates of 0.5–2% in symptomatic terms, and substantially higher rates on systematic imaging screening, documented despite universal guideline-adherent prophylaxis, reflect the failure of population-based strategies to address the high-risk tail of the hemostatic distribution. Patients in this tail—identifiable by convergent biomarker and VHA signals as described in Section 7—are currently receiving the same prophylaxis as low-risk patients and are therefore systematically undertreated. At the level of individual clinical decisions, the evidence supports the following practice-modifying conclusions. First, preoperative fibrinogen and platelet count measurement should be incorporated into the standard orthopedic trauma workup, not only to assess hemorrhagic risk (FIBTEM-MCF ≤ 11 mm, fibrinogen < 193 mg/dL), but to identify a pre-existing hypercoagulable substrate that will amplify the postoperative prothrombotic response. Second, POD-1 TAT complex measurement—or, where available, a POD-1 TEG/ROTEM assessment—provides the single highest-yield monitoring timepoint for identifying patients at imminent risk of DVT or PE, enabling early pharmacological escalation before thrombus formation occurs rather than after it is radiologically confirmed. Third, the direction of D-dimer trajectory through POD-5 to POD-7 provides a practical, low-cost longitudinal signal for monitoring the adequacy of thromboprophylaxis intensity in the post-discharge window, where clinical surveillance is most limited and VTE risk remains substantial. For patients with documented convergent high-risk hemostatic profiles—hypercoagulable VHA with concurrent TAT elevation and PAI-1 suppression of fibrinolysis—the evidence reviewed in this manuscript supports escalation from aspirin monotherapy to LMWH or DOAC, and extension of prophylaxis duration toward the 35-day threshold currently reserved by guidelines for clinically identified high-risk patients. The identification of these patients through objective hemostatic measurement, rather than clinical risk score alone, represents the translational goal toward which the research reviewed here is collectively converging.

### 8.3. Limitations of the Current Evidence Base

Several limitations of the evidence base reviewed in this manuscript must be explicitly acknowledged before clinical implementation of any patient-specific monitoring framework can be recommended with full confidence. The orthopedic-specific evidence for both biomarker-guided and VHA-guided thromboprophylaxis individualization remains predominantly observational, retrospective, and conducted in relatively small, single-center or limited multicenter cohorts. No randomized controlled trial has yet compared standard protocol-driven thromboprophylaxis against biomarker/VHA-guided individualized prophylaxis as a primary endpoint study in orthopedic trauma patients. The available meta-analyses, including the Brown et al. 2020 analysis covering 35 studies and 8939 patients, document the presence and clinical relevance of VHA-detected hypercoagulability but do not establish that VHA-guided management changes clinical outcomes [12]. This gap between diagnostic evidence and outcome-level therapeutic evidence is the most important methodological limitation constraining the transition from monitoring to management. Threshold heterogeneity constitutes a second major limitation. The MA cutoffs defining hypercoagulability range from 65 mm to 70 mm or above across studies; TAT reference ranges are assay-dependent and not universally standardized; PAI-1 thresholds have been validated in single-center hip fracture cohorts but not replicated across procedure types. The ROTEM multicenter reference ranges published by Lang et al. provide the most rigorous available normative data, but their applicability to the full spectrum of orthopedic trauma procedures—from elective arthroplasty to acute femoral neck fracture—has not been systematically validated. Finally, the absence of endothelial contribution from VHAs remains a structural limitation of viscoelastic monitoring: the in vitro whole-blood clot formed in the TEG or ROTEM cup does not replicate the in vivo thrombotic microenvironment, which is modulated by endothelial surface proteins, flow dynamics, and microparticle–endothelium interactions that are biomarker-measurable through vWF and MP-TF but not viscoelastically detectable.

### 8.4. Future Perspectives

The trajectory of the field points toward several high-priority research and translational developments that will be required to translate the diagnostic evidence base reviewed here into outcome-changing clinical practice. The most immediately needed development is a properly powered, multicenter randomized controlled trial comparing standard guideline-adherent thromboprophylaxis against a multimodal monitoring-guided individualized strategy in orthopedic trauma patients, with symptomatic VTE and major bleeding as co-primary endpoints. The design of such a trial is now feasible given the validated threshold data available from the studies reviewed, and its execution is the essential next step in establishing clinical utility—as opposed to diagnostic accuracy—for the biomarker and VHA monitoring approach described in this manuscript. Beyond the clinical trial imperative, two technological developments hold particular translational promise. Point-of-care microfluidic platforms capable of simultaneously measuring D-dimer, fibrinogen, and TAT from a single whole-blood sample alongside a VHA trace are under active development and would eliminate the logistical barriers—multiple sample types, different laboratory workflows, extended turnaround times—currently preventing concurrent multimodal monitoring from entering routine orthopedic practice. Integration of serial biomarker and VHA data into electronic clinical decision support systems, with algorithm-generated thromboprophylaxis recommendations triggered by convergent high-risk signatures, represents the pathway from monitoring evidence to bedside action at scale. The expansion of the evidence base to currently underrepresented patient subgroups is a further research priority. Pediatric orthopedic trauma, polytrauma patients with concurrent orthopedic injuries, patients on chronic anticoagulation or antiplatelet therapy, and patients with renal insufficiency all represent populations in which the biomarker and VHA thresholds established in the adult elective arthroplasty literature may not be directly applicable. Dedicated cohort studies in these subgroups are needed to define procedure-specific and comorbidity-adjusted reference ranges. Finally, the emerging role of platelet-specific VHA parameters—particularly TEG platelet mapping of the AA and ADP pathways—in tailoring antiplatelet therapy within orthopedic prophylaxis regimens deserves systematic investigation. The heterogeneity in thrombotic mechanism documented across patients—some driven by fibrin amplification, others by platelet hyperactivation, others by fibrinolytic shutdown—suggests that targeted antiplatelet or antifibrinolytic strategies, selected on the basis of individual VHA phenotyping, may offer superior risk reduction compared to the non-selective thrombin-generation-targeting approach of current LMWH and DOAC-based protocols.

### 8.5. Conclusions

Hypercoagulability in orthopedic trauma surgery is a well-characterized, mechanistically heterogeneous, and temporally predictable biological process that begins at the moment of injury or operative incision and persists, in a significant proportion of patients, well beyond the duration of standard in-hospital prophylaxis. The tools to measure this process—plasma biomarkers and viscoelastic hemostatic assays—are increasingly validated, accessible, and clinically interpretable. Their complementary integration, as outlined in this review, provides a level of hemostatic phenotype characterization that conventional coagulation tests and clinical risk scores cannot replicate. The transition from population-based to personalized thromboprophylaxis in orthopedic trauma surgery appears scientifically justified and warrants prospective clinical evaluation. Its implementation will require the randomized trial evidence currently absent from the literature, the standardization of monitoring thresholds across procedure types and patient populations, and the institutional investment in point-of-care monitoring infrastructure that the current evidence base justifies. The present review provides the conceptual framework and evidentiary foundation for that transition. Much of the current evidence originates from elective arthroplasty cohorts, highlighting the need for prospective validation in dedicated orthopedic trauma populations.

## Figures and Tables

**Table 1:** Virchow’s triad applied to orthopedic trauma surgery.

Virchow’s Component	Specific Mechanism in Orthopedic Surgery	Clinical Examples	Associated Biological Marker
Venous Stasis	Perioperative immobilization, surgical positioning, pneumatic tourniquet use, pre-existing chronic venous insufficiency	THA/TKA, hip fracture surgery, intramedullary fixation	↓ Venous flow velocity (Doppler); ↑ Postoperative D-dimer
Endothelial Injury	Surgical incision, intramedullary canal reaming, acrylic cement pressurization, release of tissue thromboplastin from bone marrow into perifemoral venous plexus	Cemented arthroplasty, intramedullary osteosynthesis, hip joint reconstruction	↑ vWF; ↑ Tissue Factor (TF); ↓ Thrombomodulin; ↑ Factor VIII
Hypercoagulability	Extrinsic pathway activation (TF/FVIIa complex), release of pro-inflammatory cytokines (IL-6, TNF-α), fibrinolysis suppression (↑ PAI-1), consumption of natural anticoagulants (ATIII, Protein C)	Polytrauma, femur fracture, major orthopedic surgical procedures	↑ TAT, ↑ F1+2, ↑ Fibrinogen, ↑ D-dimer; TEG: ↓ R time, ↑ MA; ROTEM: ↓ CT, ↑ MCF

Legend: THA = total hip arthroplasty; TKA = total knee arthroplasty; TF = tissue factor; vWF = von Willebrand factor; PAI-1 = plasminogen activator inhibitor-1; ATIII = antithrombin III; TAT = thrombin–antithrombin complexes; F1+2 = prothrombin fragments 1+2; MA = maximum amplitude (TEG); MCF = maximum clot firmness (ROTEM); CT = clotting time (ROTEM); IL-6 = interleukin-6; TNF-α = tumor necrosis factor alpha.

**Table 2:** VTE risk factors in orthopedic trauma: Classification, mechanism, clinical consequence, and relevant biomarker.

Risk Factor	Pathophysiological Mechanism	Clinical Consequence	Relevant Biomarker
Hip fracture	Massive soft tissue injury + TF release from trabecular bone + immobilization-induced stasis	Proximal DVT risk 10–20% without prophylaxis	↑ D-dimer, ↑ TAT, ↑ F1+2
Total knee arthroplasty (TKA)	Pneumatic tourniquet → accentuated stasis; acrylic cement → fat embolism and TF release; knee flexion → popliteal vein compression	Distal DVT frequent (15–40% without prophylaxis); moderate PE risk	↑ D-dimer, ↑ Fibrinogen; TEG: ↑ MA
Total hip arthroplasty (THA)	Intramedullary pressurization → thromboplastin release into perifemoral venous plexus; direct endothelial injury	Proximal DVT + higher PE risk compared to TKA	↑ vWF, ↑ Factor VIII, ↑ TAT
Advanced age (>65 years)	Chronic endothelial dysfunction, reduced t-PA activity, elevated baseline PAI-1, cardiovascular comorbidities	VTE risk 2–3× higher than younger patients	↑ Baseline D-dimer, ↑ Fibrinogen, ↑ vWF
Obesity (BMI > 30)	Venous stasis from mechanical compression, chronic pro-inflammatory state (↑ adipocyte-derived IL-6), prolonged postoperative immobilization	VTE risk proportional to BMI; anticoagulant dosing challenges	↑ PAI-1, ↑ D-dimer, ↑ Fibrinogen
Hereditary thrombophilia	Factor V Leiden mutation, Protein C/S deficiency, prothrombin G20210A mutation → resistance to natural anticoagulants	Recurrent or early-onset postoperative thrombotic episodes	TEG: ↑ MA, ↓ R time; ROTEM: ↑ MCF
Prolonged immobilization	Absence of muscle pump → stasis in deep veins of lower extremities	Asymptomatic DVT, predominantly at calf level	↓ Doppler flow velocity; ↑ D-dimer
Polytrauma (ISS > 16)	Massive coagulation activation via DAMPs, cytokines, TF; surgical “second-hit” phenomenon	Consumptive coagulopathy → paradoxical thrombosis or hemorrhage	↑ IL-6, ↑ TNF-α, ↑ TAT, ↑F1+2; TEG/ROTEM: variable profile

Legend: THA = total hip arthroplasty; TKA = total knee arthroplasty; TF = tissue factor; vWF = von Willebrand factor; PAI-1 = plasminogen activator inhibitor-1; TAT = thrombin–antithrombin complexes; F1+2 = prothrombin fragments 1+2; MA = maximum amplitude (TEG); MCF = maximum clot firmness (ROTEM); ISS = Injury Severity Score; DAMP = damage-associated molecular patterns; IL-6 = interleukin-6; TNF-α = tumor necrosis factor alpha; t-PA = tissue plasminogen activator; DVT = deep vein thrombosis; PE = pulmonary embolism; BMI = body mass index. Elective total hip arthroplasty (THA) and total knee arthroplasty (TKA) are included because they represent the best-studied orthopedic models of perioperative hypercoagulability and biomarker performance, although they are distinct from orthopedic trauma.

**Table 3:** Coagulation biomarkers in orthopedic trauma surgery: Mechanisms, optimal sampling window, diagnostic performance, and limitations.

Biomarker	Biological Mechanism	Phase of Hemostasis Reflected	Optimal Sampling Window	Key Clinical Data in Orthopedic Surgery	Reported Cutoff/AUC	Limitations in Perioperative Setting
D-dimer	Plasmin-mediated degradation product of cross-linked fibrin	Fibrinolysis (downstream)	POD 1 + POD 7 (bimodal monitoring)	Universal elevation post-THA/TKA (92–100% exceed standard threshold); diagnostic yield improves when combined with fibrinogen in patients >60 years	Standard: 0.5 mg/L; Age-adjusted: age × 0.02 mg/L; AUC 0.73	Very low specificity postoperatively; near-universal elevation masks true thrombotic events; requires age and procedure-specific adjustment
Fibrinogen	Acute-phase protein; primary substrate for thrombin-mediated fibrin clot formation; determinant of clot mechanical strength	Coagulation substrate + acute-phase response	Preoperative baseline + POD 3–5 (inflammatory peak)	OR 2.09 for DVT in intertrochanteric fracture [11]; specificity 0.777, superior to D-dimer alone; combination with D-dimer improves PPV in patients >60 years	Cutoff: 3.543 g/L; Specificity 0.777	Low sensitivity as standalone marker; confounded by inflammatory states, malnutrition, liver disease, pregnancy
TAT (Thrombin–Antithrombin complexes)	Formed when active thrombin is neutralized by ATIII; direct real-time quantitative marker of in vivo thrombin generation	Thrombin generation—central coagulation axis	POD 1 (critical early predictive window)	TAT on POD-1 predicts DVT on POD-7 after THA/TKA; nomogram incorporating TAT achieved AUC 0.836–0.957, significantly outperforming Caprini score [14,15]	Normal range: 0.00–0.55 mg/L; procedure-specific cutoff not yet universally validated	Very short plasma half-life; requires rapid sample processing; no standardized orthopedic-specific reference intervals
F1+2 (Prothrombin Fragments 1+2)	Released stoichiometrically during prothrombin → thrombin conversion by prothrombinase complex (FXa/FVa); upstream cumulative thrombin generation marker	Thrombin generation—upstream/cumulative	Day of surgery + POD 1 + POD 6 (sustained activation window)	Significantly elevated through POD 6 in THA patients, indicating sustained thrombin activation beyond the operative window; positive correlation with TAT; also measurable in urine	Plasma half-life ~90 min; urinary excretion detectable for several days postoperatively; no validated orthopedic cutoff	Requires specialized ELISA; limited routine clinical availability; plasma and urine levels show poor correlation in late postoperative period
PAI-1 (Plasminogen Activator Inhibitor-1)	Principal inhibitor of t-PA and urokinase → suppresses plasmin generation and fibrinolysis; upregulated by surgical stress and IL-6	Fibrinolysis inhibition	POD 1 (highest predictive value window)	PAI-1 > 53.5 ng/mL on POD-1: sensitivity 100%, specificity 67% for VTE prediction after THA under mechanical prophylaxis only; combined with soluble fibrin: negative agreement rate 98%	Cutoff: 53.5 ng/mL (POD-1, THA, mechanical prophylaxis only)	Predictive value attenuated or abolished by pharmacological anticoagulation; limited data outside THA; not available in routine clinical laboratory panels
vWF (von Willebrand Factor)	Large multimeric glycoprotein released from endothelial Weibel–Palade bodies upon injury or inflammatory stimulation; mediates platelet adhesion to subendothelium; stabilizes and carries FVIII	Endothelial activation + primary hemostasis	Intraoperative (↓ consumptive decrease) + POD 1 (↑ acute-phase rise)	Postoperative FVIII rise up to 4.5 IU/mL on POD-1 after elective orthopedic surgery; FVIII > 2.30 IU/L independently associated with VTE risk in population studies	FVIII > 2.30 IU/L: independent VTE risk factor; no validated vWF-specific orthopedic threshold	Reflects endothelial activation but not clot formation directly; influenced by ABO blood type, age, inflammatory state, and estrogen levels
PT/aPTT/INR (standard tests—listed for comparison)	Assess isolated clotting pathway activity under cell-free, non-physiological, platelet-poor plasma conditions	Isolated single-pathway assessment only; no whole-blood dynamics	Standard perioperative laboratory monitoring	Systematically insensitive to hypercoagulable states; normal values documented even in patients who subsequently develop DVT or PE; do not capture platelet contribution, fibrinolysis, or clot viscoelastic properties	N/A—designed to detect factor deficiency, not excess activation	Cannot detect hypercoagulability; normal results provide false reassurance; do not reflect perioperative thrombotic risk in orthopedic patients

Legend: POD = postoperative day; TAT = thrombin–antithrombin complexes; F1+2 = prothrombin fragments 1+2; PAI-1 = plasminogen activator inhibitor-1; vWF = von Willebrand factor; ATIII = antithrombin III; t-PA = tissue plasminogen activator; FVIII = coagulation factor VIII; ELISA = enzyme-linked immunosorbent assay; AUC = area under the ROC curve; PPV = positive predictive value; OR = odds ratio; THA = total hip arthroplasty; TKA = total knee arthroplasty; DVT = deep vein thrombosis; VTE = venous thromboembolism; PE = pulmonary embolism; IL-6 = interleukin-6. Several biomarker thresholds summarized in this table originate from elective THA/TKA studies because equivalent trauma-specific validation studies remain limited.

**Table 4:** Proposed multimodal monitoring framework for perioperative hypercoagulability in orthopedic trauma surgery.

Timepoint	Recommended Biomarkers	Recommended VHA	Key Thresholds	Clinical Action
Preoperative	Fibrinogen, D-dimer (age-adjusted), platelet count	TEG (MA, CI) or ROTEM (EXTEM/FIBTEM MCF)	Fibrinogen > 3.543 g/L; Platelet ≥ 277 × 10^9^/L; FIBTEM-MCF ≤ 11 mm (hemorrhagic risk); MA > 65 mm	Consider DOAC/LMWH over aspirin in high-risk patients; flag low FIBTEM-MCF for blood-sparing preparation
Intraoperative	—	TEG (R, K, α, MA, CI) if available	CI > +3.0; MA > 66 mm; α-angle elevation	Document intraoperative hypercoagulable phenotype; initiate prophylaxis as early as surgical hemostasis permits
POD-1	TAT complexes, PAI-1, fibrinogen	TEG/ROTEM (MA/MCF, CI, Ly30/LI30)	TAT > 0.55 mg/L FEU; PAI-1 > 53.5 ng/mL; MA > 66.7 mm or CI > +3.0	High-risk convergent profile: initiate DOAC or LMWH; consider extended duration; add mechanical prophylaxis
POD-3	—	ROTEM (EXTEM-MCF, INTEM-A10)	EXTEM-MCF ≥ 66 mm	Reassess prophylaxis adequacy; consider imaging (duplex ultrasound) in asymptomatic high-risk patients
POD-5 to POD-7	D-dimer (age-adjusted), fibrinogen	TEG/ROTEM if clinically indicated	D-dimer rising trajectory; fibrinogen > 3.543 g/L persisting	Evaluate for subclinical DVT; reassess anticoagulation duration; consider extending prophylaxis to 35 days

Legend: TAT = thrombin–antithrombin complexes; PAI-1 = plasminogen activator inhibitor-1; VHA = viscoelastic hemostatic assay; FIBTEM = fibrinogen-specific ROTEM assay; EXTEM = extrinsic ROTEM assay; INTEM = intrinsic ROTEM assay; MA = maximum amplitude; MCF = maximum clot firmness; CI = coagulation index; Ly30 = lysis at 30 min; LI30 = lysis index at 30 min; DOAC = direct oral anticoagulant; LMWH = low-molecular-weight heparin; POD = postoperative day; DVT = deep vein thrombosis. Thresholds shown in this table are derived from individual studies discussed in Section 4, Section 5 and Section 6 and are presented as research-informed examples rather than universally accepted clinical decision limits. Most require prospective validation in large orthopedic trauma cohorts before routine clinical implementation. None of the proposed thresholds should be interpreted as validated clinical decision criteria for orthopedic trauma patients.

## Data Availability

No new data were created or analyzed in this study. Data sharing is not applicable to this article.

## Abbreviations

The following abbreviations are used in this manuscript:

A10	Amplitude at 10 min
aPTT	Activated Partial Thromboplastin Time
AT	Antithrombin
CI	Coagulation Index
CT	Clotting Time
DOAC	Direct Oral Anticoagulant
DVT	Deep Vein Thrombosis
EXTEM	Extrinsic ROTEM Assay
FEU	Fibrinogen Equivalent Units
F1+2	Prothrombin Fragment 1+2
FIMBTEM	Fibrin-Based ROTEM Assay
INR	International Normalized Ratio
INTEM	Intrinsic ROTEM Assay
IL-6	Interleukin-6
K	Clot Kinetics Time
LI30	Lysis Index at 30 min
LMWH	Low-Molecular-Weight Heparin
LY30	Clot Lysis at 30 min
MA	Maximum Amplitude
MCF	Maximum Clot Firmness
PAI-1	Plasminogen Activator Inhibitor-1
PE	Pulmonary Embolism
POD	Postoperative Day
PT	Prothrombin Time
R	Reaction Time
RCT	Randomized Controlled Trial
ROTEM	Rotational Thromboelastometry
TAT	Thrombin–Antithrombin Complexes
TEG	Thromboelastography
THA	Total Hip Arthroplasty
TKA	Total Knee Arthroplasty
VHA	Viscoelastic Hemostatic Assay
VTE	Venous Thromboembolism
vWF	von Willebrand Factor
